# Innovative outpatient treatment for veterans and service members and their family members

**DOI:** 10.3389/fpsyt.2024.1377433

**Published:** 2024-07-24

**Authors:** Gabrielle Groth Hoover, Andrew Teer, René Lento, Peter Ward, Rebecca J. Zakarian, William Tinney, Wesley Sanders, Katrina Echevarria, Joseph Bonvie, Kathleen Dunford, Jessica Covitz, Kaloyan S. Tanev

**Affiliations:** Massachusetts General Hospital, Department of Psychiatry, Boston, MA, United States

**Keywords:** veterans, service members, mental health care access, psychiatry, innovation

## Abstract

In 2009, Massachusetts General Hospital and the Red Sox Foundation launched Home Base, a nonprofit dedicated to providing care to veterans, service members, and their loved ones who struggle with the invisible wounds of war free of charge. Significant needs exist for mental health services in each of these populations, and a need for innovative approaches to address shortcomings in existing treatment models. Three inventive components of our programming are highlighted herein: a Veteran Outreach Team, which helps to engage patients in care, programming, and services specifically for family members, and an intensive outpatient substance use treatment program. More than 4,000 patients, 3,031 veterans and service members, and 1,025 family members have engaged in treatment at Home Base. Patients were asked to complete post-treatment self-measures, including a satisfaction questionnaire via an electronic data collection system. The vast majority of individuals who engaged in our treatment model were satisfied with the care they received (>92%) and would refer their peers to the Home Base program (>75%). Data from 78 individuals who completed the dual diagnosis services demonstrated large effect sizes in reductions in alcohol use and comorbid mental health symptoms. These data suggest that novel components to the standard outpatient mental health model might provide substantive benefits for the patients served. While internal data is prone to a lack of generalizability, these additional offerings help ameliorate patients’ expressed shortcomings with existing models; present literature that describes the benefits that these additions provide is also reviewed. The lessons learned and limitations are discussed.

## 1 Introduction

More than 18 million veterans are living in the United States, accounting for approximately 6% of the adult population (1). Veterans frequently present with complex medical and mental health concerns when they do seek care. Recent prevalence estimates suggest that 9.4% of veterans develop posttraumatic stress disorder (PTSD) in their lifetime (2). The prevalence rate of traumatic brain injury (TBI) for post-9/11 veterans has been estimated to be around 20% (3), and one meta-analysis evinced a prevalence rate for co-morbid PTSD of around 37% in veterans who have sustained TBI (4). The ripple effect of these concerns on veteran’s family members is also substantial (5, 6), and rates of spousal psychiatric conditions are similarly high, with over 35% of military spouses screening positive for a psychiatric diagnosis in one large study of over 9,000 military-affiliated couples (7). Barriers to care for veterans and their family members are numerous and can include stigma, lack of information about where and how to access care, concerns about privacy, difficulty navigating healthcare systems, and ineligibility for Veterans Healthcare Administration (VHA) services based on discharge status. Additional practical barriers often exist, such as transportation, logistics, or time away from work, which can be prohibitive for some veterans particularly in rural areas (8, 9). Cultural emphasis on self-reliance among military service members may also contribute to reluctance to access mental health care (10).

**Figure 1:**
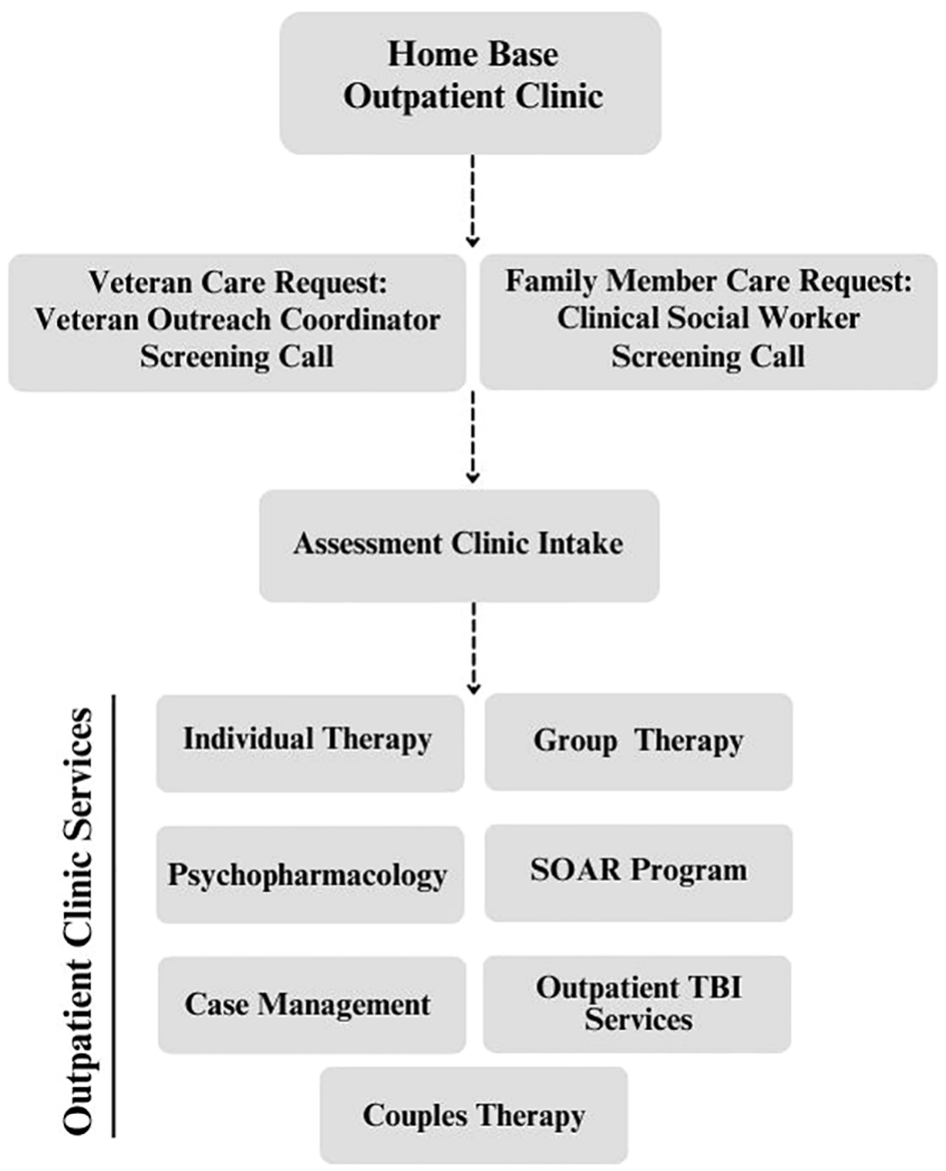
Flowchart overview of Home Base Outpatient Clinic Processes.

Only 48% of veterans utilize any single VHA (Veteran’s Health Administration) service and an even smaller percentage –around 20% – use it as their primary source of health care (9, 11). Among Massachusetts veterans, only 36% were enrolled in VA healthcare, while only 61% of those enrolled received care according to a 2017 Assessment of Needs, Well-Being, and Available Resource (RAND; (12)). Despite the low rates of service utilization, this needs assessment also found that current National Guard and Reserve members had high rates of depression, PTSD, and binge drinking. Furthermore, the same needs assessment evinced unmet needs related to family services. There are various obstacles and barriers to accessing mental health services, some of which can impede timely access to care for veterans or service members as well as family members (13). Given these concerns regarding mental health care accessibility and utilization in Massachusetts, Home Base was purposefully established to fill this void. Please see **Figure 1** for a visual overview of the clinic programming.

Moreover, while solutions exist for many of these barriers to care, our team isolated three that seemed ameliorable through concerted changes in our clinic’s structure and operations. First, according to the most recent systematic review of the Veterans Affairs’ (VA) mental health services, the majority of veterans reported being dissatisfied with the mental health resources at their local Veterans Affairs Medical Center, in addition to concerns of detrimental consequences of utilizing those mental health services (e.g., distrust in VA, repercussions with current job; 14). Similarly, qualitative research has demonstrated that apprehensions about providers’ lack of insights regarding veterans’ lived experiences, difficulty connecting with providers, and invalidating interactions often lead veterans to drop out and achieve suboptimal treatment responses (14). With these findings in mind, our clinic was set up so that veterans on staff serve as points of contact throughout the patients’ treatment journeys.

Second, in veteran populations, close relationships are often both drivers of initiating treatment when the symptoms worsen (15) and factors that support higher functioning (16). Family-related concerns ranging from functional (e.g., arranging childcare) to legal (e.g., concerns of losing custody/visitation) to psychosocial (e.g., straining relationships and losing respect) create obstacles for veterans and service members (17). To leverage relationships as protective factors and to address the mental health concerns faced by family members, Home Base has a family program that provides psychoeducation and therapy (individual, group, and couples).

Third, the prevalence of both PTSD and a co-occurring substance use disorder among US adults has been estimated at 30-40% (18, 19). Dropout among dual diagnosis patients, especially from frontline trauma-focused treatment, remains a significant problem (20–22). Moreover, literature has linked substance use with avoidance, which decreases the effectiveness of trauma-focused treatments (23). Therefore, our team established a group-based intensive outpatient program for substance use to address these concerns, often delivered concurrently with other mental health services in line with current best practices.

### 1.1 Veteran outreach team

Within the military community, a culture of camaraderie shared among service members is widely seen as critical to unit cohesion and mission readiness (24). In one qualitative report, veterans themselves identified that shared bonds commonly serve as a facilitator of care, such that individuals receiving encouragement from their peers may experience a reduction in stigmatized beliefs around mental health care and be more likely to seek treatment (25). Peer support models have been shown to leverage the strengths of the veterans’ shared military experience to decrease barriers to care and increase engagement with mental health treatment (26, 27). Past research of Home Base’s peer support model has demonstrated that the veteran outreach coordinator (VOC) team significantly improves treatment engagement such that veterans receiving contact from VOCs attend more sessions of therapy and are evinced a 17% lower drop-out rate (28). This improvement is noteworthy given that service members and veterans face stigmatized beliefs about seeking mental health treatment to begin with (29, 30).

### 1.2 Family programming

With an estimated 47.6% of military service members married, the approximately 2.6 million family members of military service members, to include their 1.6 million children, are a critical consideration when assessing the family unit’s need for mental health services (31). Parents of military service members also often serve as important supports during and after their service, with many veterans returning to live with their parents during the transition to civilian life (32, 33). Although family members display resilience to the stressors of military life (e.g., frequent relocations), these stressors can increase the risk to the mental health of family members (34–36). After the end of the service member’s career and their transition to veteran status, they commonly experience mental health difficulties, such as PTSD, depression, and substance use disorders which further impact their family unit (e.g., parenting dysfunction, relationship dysfunction; (37, 38)). These difficulties are compounded by the loss of resources previously available during military service (39). Importantly, past research indicates that family-related mental health challenges may have a reciprocal effect on the veteran’s mental health, such that changes in one domain can have corresponding effects in others (40, 41). Thus, addressing family members’ mental health difficulties can be beneficial to the veteran’s mental health, and vice versa. Past research also indicates that family members can influence their Veteran’s mental health service use (17). While the importance of providing care to the veteran’s family has been recognized by the VHA (42), these services are only accessible to families with a veteran actively receiving care, in specific circumstances, and where available.

### 1.3 Skills-based outpatient addiction recovery (SOAR)

Substance use disorders (SUDs) are frequently comorbid with PTSD and other mental health conditions (43, 44), and established literature supports the concurrent treatment of these conditions (45). Further, individuals with substance use disorders (even when not diagnosed with a co-occurring mental health disorder) have higher odds of accessing treatment for mental health than treatment for SUD (46), potentially due to the greater normalization in the United States of seeking mental health treatment compared to substance use treatment (47). Accordingly, epidemiology studies have encouraged general mental health providers to develop proficiency in diagnosing and treating SUDs to increase accessibility and reduce the stigma associated with substance use treatment (46). To meet this need evident in population the Home Base Program serves, SOAR employs intensive outpatient group therapy program for SUDs and co-occurring mental health conditions that utilizes harm reduction principles.

## 2 Context

Massachusetts General Hospital and the Red Sox Foundation launched the nonprofit Home Base Program in 2009, as a public-private partnership and the first and largest private sector clinic for Veterans and service members in the nation. The programming provided has expanded substantially over the past 15 years while the mission has remained the same: to provide mental health care at no cost to veterans, service members, and their families. Additionally, care is provided to veterans of all eras, regardless of deployments, branch, and discharge status, which encompasses a large population of Massachusetts. The 2022 American Community Survey estimated there are 266,304 Veterans living in the Commonwealth of Massachusetts (48). This figure, however, does not include National Guard or Reserve component members who were not called or activated to federal service, nor service members residing in Massachusetts, which is home to Coast Guard, Army, and Air Force Bases. Since the start of the COVID-19 pandemic, services in the outpatient clinic have been conducted in a hybrid manner with the majority of therapy sessions (group and individual) and medication management appointments, occurring via synchronous telehealth sessions. Telehealth sessions have routinely demonstrated effectiveness, especially as the delivery means for trauma-focused therapy [e.g. (49)] and have the benefit of reducing some barriers to accessing care [e.g. (50)].

## 3 Key programmatic elements

### 3.1 Veteran outreach team

Our VOC team conducts outreach throughout a diverse range of communities to strategically connect with service members and Veterans. The objective is to forge trust by leveraging our cultural competency and to educate others on how our tailored clinical programming can serve the needs and interests of local service members and Veterans. Additionally, the VOC team engages key stakeholders (e.g., local Veteran Service Officers, leaders at military-related non-profits) within our mental health ecosystem to develop community partnerships that extend the reach of our message: Home Base is a lifeline for Veterans and service members in need.

In filling this unique position, the VOC team serves as the “front door” to the Home Base program, providing initial screenings for services and assisting Veterans in getting connected to care. This creates substantive benefits and distinct challenges. On one hand, the shared background of service is designed to enhance rapport building from the outset. To this end, our VOC team is purposefully comprised of veterans across the Armed Forces with diverse backgrounds and experiences (e.g., job positions). Indeed, internal data from patient feedback surveys reinforce this benefit and demonstrate that the VOC team’s presence serves to increase trust and reassure the Veteran that their needs are understood. On the other hand, this creates a noteworthy challenge: VOC staff are not trained clinicians but often encounter clinical challenges (e.g., risk issues) during their patient-facing work in our clinic. VOCs are trained to facilitate screenings and collaborate with individuals’ care teams to manage these concerns; VOCs have biweekly consultations with a licensed clinician to discuss and troubleshoot challenging cases. In sum, the VOC team leverages their shared military service to navigate and interpret how participants talk about their service, military jobs, deployments, traumas, military culture, and other factors relevant to meeting the needs of the individual.

### 3.2 Family Programming

The Home Base Outpatient Clinic model provides a multi-disciplinary care approach for adult (18 years old or older) family members of veterans and service members. This program’s primary focus is to help family members to better understand their family members’ mental health concerns and to address mental health difficulties they may experience as a result of their family member’s military service and/or mental health concerns. Family programming is offered in individual, group, and couples formats, with most care currently offered via telehealth. Furthermore, “family” is defined broadly to include a wide range of supportive relationships, not limited to biological family, allowing any family member experiencing mental health difficulties related to their family member’s military service to seek care regardless of their veteran’s engagement in care.

For individual and group services, family members’ care begins with a brief phone screen followed by a 90-minute diagnostic mental health evaluation. An intake clinician then offers the family member recommendations tailored to their concerns. Individual interventions offered include short-term, evidence-based therapies such as psychoeducation on PTSD and co-occurring conditions, cognitive-behavioral therapies, and Community Reinforcement and Family Training (CRAFT) for families impacted by substance use (51). Medication management services are also offered to family members when indicated. Group interventions include a 10-week Family Skills group comprised of cognitive behavioral skills and a 12-week CRAFT group. Parenting interventions are additionally offered as a 7-week Skillful Parenting education course and in a consultation format (i.e., 1-3 sessions in an individual or conjoint structure). Case management services are available to family members as part of their care which assists with care coordination, identifying psychosocial resources, and referrals to community providers for services not available at Home Base. Case management referrals also assist with referrals to pediatric services for family members under the age of 18 when indicated or requested.

Couples therapy begins with each partner completing a phone screen followed by a four-session assessment. The couples assessment includes a conjoint session, individual sessions with each partner, and a feedback session, during which the therapist discusses treatment recommendations. Modalities offered for couples therapy include emotion focused therapy for couples and integrative behavioral couple therapy.

### 3.3 SOAR

The SOAR program patients is an intensive outpatient group therapy program for SUDs and co-occurring mental health conditions. The SOAR program aims to overcome frequent barriers to program participation and completion by (a) not requiring abstinence to participate, and (b) operating within a general mental health clinic rather than in a substance use specialty clinic. Having launched in a fully virtual format in 2022, SOAR patients enter Home Base through the general outpatient clinic and subsequently complete a SOAR orientation with a clinician point-of-contact (POC) to set personalized goals (e.g. abstinence or harm-reduction) and commit to a 4–8-week episode of care. The SOAR program consists of three 90-minute groups per week. Group sessions emphasize cognitive behavioral therapy and mindfulness-based relapse prevention skills. The program also incorporates adjunctive material from Seeking Safety (52), dialectical behavior therapy, and acceptance and commitment therapy. Participants meet with their SOAR POC at least three times during the program to address further goal setting, treatment planning, and barriers to care and engagement. SOAR patients may also access any other services offered through the Home Base, such as medication and individual therapy.

## 4 Methods

Home Base has gathered survey data on pre- and post-treatment outcomes for patients seeking care in the outpatient clinic since its inception. The outcome measures collected have changed over time as staff members request to add and remove items based on research interests, creating an inconsistent dataset over time. Prior to 2020, when the vast majority of care was provided in person, measures were collected while patients waited for appointments using computer stations and/or tablets provided by Home Base. Since 2020, with the vast majority of care now being provided virtually, measures have been collected via links to secure online surveys emailed to patients. The completion rate of measures tends to be low (i.e., 34% in 2023-2024), although steps being taken to address this concern are noted in later sections.

Starting in 2021, an annual satisfaction survey has been sent to outpatients (i.e., both veterans/service members and their loved ones) to gather data on their satisfaction with Home Base services. It was originally designed to gauge satisfaction with virtual care in the context of the pandemic and has been administered each year as it provides helpful feedback regarding our provision of services. Given the focus of the current paper, the consistent data collection process of the satisfaction survey compared to the outcome measures, and the similar response rate (i.e., 30%), the methods and results of the annual satisfaction survey are highlighted. Likert scale items (e.g., “Overall I feel satisfied with the care I’ve received at Home Base” with five response options ranging from Strongly Disagree to Strongly Agree), categorical questions (e.g., “What brought you to Home Base?” with various response options including Anxiety, Depression, Family or Loved One Encouraged Me to Get Care) and open ended items (e.g., “What do you like best about Home Base?” “What do you like least about Home Base?”) are included.

The SOAR program collects data utilizing a more targeted approach; SOAR participants complete pre-treatment measures that include the PHQ-9 (53), the BAM (54), and What I Want From Treatment (55) as well as post-treatment measures that include the PHQ-9, the BAM, and What I Got From Treatment, and several measures of patient satisfaction. While measures are sent via email, the patients’ point of contact provider and the clinical research coordinator supporting the program provide specific follow-up reminders to patients to have them complete the survey, often during clinical encounters in the virtual IOP.

## 5 Results

The outpatient clinic at Home Base has served more than 4,000 patients: 3,031 veterans/service members and 1,025 family members since its inception in 2009, with the number of patients seeking care growing year over year. In 2023, the clinic provided care to 656 veterans/service members and 108 family members as compared to 513 veterans/service members and 78 family members in 2022.

Ninety-seven veterans and service members participated in our most recent (2023) survey of patient satisfaction. Among these respondents 76% (N = 71) identified as male and 19.6% (N = 19) reported being 18-34 years old; more than half (i.e., 52.6%, N = 51) reported being between 35-51 years old, and 15.5% (N = 15) reported being 52-68 years old. The respondents identified their branch of service as follows: Air Force (11.7%, N = 11), Army (44.7%, N = 42), Coast Guard (10.6%, N = 10), Marine Corps (17%, N = 16), and Navy (13.8%, N = 13). 85.9% (N = 79) of respondents identified as white, 1% (N = 1) as Asian, 3.3% (N = 3) as black or African American, and 5.4% (N = 5) as multiracial. 84.8% (N = 78) of respondents identified as non-Hispanic or non-Latinx.

**Figure 2:**
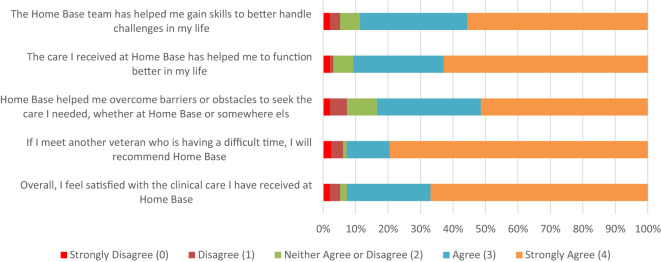
Veteran Responses to Home Base Outpatient Clinic 2023 Satisfaction Survey (N = 97).

The majority of responders (92.8%, N = 90) agreed or strongly agreed with the statement, “Overall, I feel satisfied with the clinical care I have received at Home Base.” To the prompt “If I meet another veteran who is having a difficult time, I will recommend Home Base,” 79.3% (N = 77) chose “strongly agree” or “agree.” For the item “Home Base helped me overcome barriers or obstacles to seek the care I needed, whether at Home Base or somewhere else,” 83.5% (N = 81) answered “agree” or “strongly agree.” Some of the written feedback from veterans and service members in response to the question “What do you like best about Home Base?” included “A staff that truly cares and I can feel it.” “Home Base should be the standard for all treatment of veterans. I really feel the staff really know what they are doing and get the most help for patients.” and “The care is phenomenal.” See **Figure 2** for a visual of veteran Satisfaction Survey responses.

Drawing from the same (2023) satisfaction survey, 100% (N = 14) of family respondents identified as female, 93% (N = 13) identified as white, and 86% (N = 12) identified as non-Hispanic/Latinx. The age of family members varied: 29% (N = 4) reported being 18-34 years old, 29% (N = 4) reported being 35-51 years old, and 29% (N = 4) noted being 52-68 years old. The majority of family respondents identified as the partner of a veteran (64%, N = 9), 7% (N = 1) as the sibling of a veteran, 14% (N = 2) as a dependent child, and 14% (N = 2) as the parent of a veteran.

**Figure 3:**
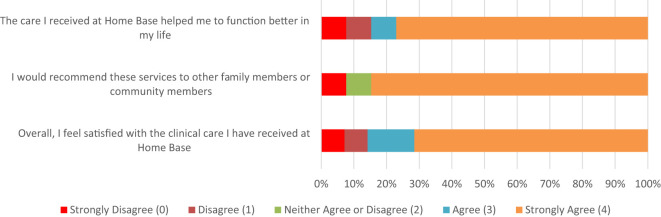
Family Responses to Home Base Outpatient Clinic 2023 Satisfaction Survey (N = 14).

Nearly 86% (N =12) of family members chose “strongly agree” or “agree” in response to the prompt: “Overall, I feel satisfied with the clinical care I have received at Home Base.” When asked whether they would recommend Home Base services to other family members or community members, 79% (N = 11) of respondents strongly agreed. Family members responding to the question “What do you like best about Home Base?” wrote: “The mission of the program, the support from all team members, the professionalism of the clinicians.” and “Genuinely caring and professional clinicians.” See **Figure 3** for a visual of Family Satisfaction Survey responses.

Since its inception in 2019, the virtual SOAR program has served 78 unique patients, the majority (N = 58, 74.4%) of whom self-selected an abstinence goal as their primary treatment target and a sizeable minority opted for a reduction goal (N = 20). The SOAR completion rate has been 67%. The majority sought care for alcohol use; additional presenting concerns included cannabis, stimulants, opioids, gambling, and pornography use. Outcome data are displayed in **Table 1**; outcome data demonstrate statistically significant reductions in alcohol use (d = 0.82, p =.002), other substance use (d = 0.44, p =.044), and depression symptoms (d =.72, p =.006) among those with an abstinence goal. Patients with abstinence and reduction goals both indicated increased satisfaction with progress toward their recovery goals at post-treatment (d = -0.82, p =.002; d = -0.96, p =.050).

**Table 1:** Skills-based Outpatient Addiction Recovery (SOAR) Program Outcome Data.

		*M diff*	*SD*	*t*	*df*	*p*	Cohen’s *d*
Abstain	Alcohol use	0.94	1.14	3.39	16	.002*	0.82
	Binge alcohol use	1.00	1.27	1.94	5	.055	0.79
	Other substance use	0.47	1.07	1.82	16	.044*	0.44
	Satisfaction with recovery progress	-0.94	1.14	-3.39	16	.002*	-0.82
	PTSD symptoms	5.33	13.66	0.96	5	.191	0.39
	Depression symptoms	3.43	4.80	2.86	15	.006*	0.72
Reduce	Alcohol use	1.00	1.16	1.73	3	.091	0.87
	Binge alcohol use	1.33	1.16	2.00	2	.092	1.16
	Other substance use	-0.80	1.79	-1.00	4	.187	-0.45
	Satisfaction with recovery progress	-0.80	0.84	-2.14	4	.050*	-0.96
	Depression symptoms	2.50	2.52	1.99	3	.071	0.99

*p <.05. PTSD, posttraumatic stress disorder. Substance use and recovery variables were measured using the Brief Addiction Monitor (BAM); PTSD symptoms were measured using the PTSD Checklist for DSM-5 (PCL-5); depression symptoms were measured using the Patient Health Questionnaire (PHQ-9). PTSD data not available for the Reduce group.

## 6 Discussion

The Home Base Outpatient Clinic is an innovative clinical program designed to address the complex mental health needs of veterans, service members, and their family members by reducing and/or mitigating common barriers to care in addition to enhancing the availability of needed multidisciplinary services. There are three innovative elements of our clinic that we have described in this manuscript. First, Home Base’s unique utilization of a Veteran Outreach Team to facilitate patient engagement in care is one innovative structural element that leverages military culture, social support, and shared community bonds to reduce stigma associated with seeking mental health care services. VOCs are involved in the program planning process alongside clinical leadership, which allows them to offer their insight on military culture and community considerations. This results in clinical programs and care that are culturally competent. In line with past research on the impact of peer support on recovery (27, 28), our internal surveys indicate the VOC team’s support benefits treatment outcomes through shared understanding and increased retention of our patients.

Second, family programming is another innovative element of our clinic. It provides multidisciplinary services to individuals who are often in need of but less able to access military-informed care. The suffering of service members affects their families, and resilient families improve the functional outcomes of service members carrying invisible wounds of war (41, 56). Additionally, when family members seek care with the clinic before their veteran or service member is engaged in care, they can build trust and familiarity with mental health care and bring their loved ones into care. Additionally, previous research completed at Home Base has demonstrated that engagement in couples therapy and parenting interventions can help build motivation and commitment in the veteran to engage in individual treatment to address underlying individual mental health concerns contributing to relational stress (56). On the provider side, integrating care for family members within the same clinic as their veterans and service members offers opportunity for collaborative care between team providers. Previous research with military families has shown that when a family is engaged in both couples and individual therapy modalities, individual and couples therapy providers can understand presenting concerns more deeply within a family systems framework (57). In annual satisfaction surveys, family members often express gratitude for the opportunity to access care that considers the impact of their family member’s service on the family’s well-being with clinicians who have a strong understanding of military culture.

Third, the SOAR program fills an important need of providing intensive outpatient substance use services within a larger mental health clinic setting. Initially, patients presenting with SUD were referred to external IOP programs for services before becoming eligible for services within Home Base. This refer-out is suboptimal and provides additional opportunities for patients seeking services to fall out of care. The comorbidity between PTSD and SUD and the need for available, military-culture competent care was great enough to warrant starting the SOAR program to address these needs. Data from patients support the feasibility and efficacy of providing virtual, group-based treatment for patients with co-occurring mental health and substance use presentations, as well as for patients with mixed reduction and abstinence goals.

Overall, the abovementioned three programmatic components of the Home Base Outpatient Clinic have been integral to the positive outcomes we have evinced in helping veterans, service members, and their families connect to and engage in impactful mental healthcare. Our satisfaction survey results have borne out this positive impact with many respondents commenting on the “Veterans helping other veterans,” the “welcoming and helpful” nature of the staff, and the “Veterans on staff” being their favorite part of the program. Family members echo these sentiments commenting on the “comprehensive services,” “feeling understood,” and “attended to.”

In sum, implementing this innovative approach may be difficult to replicate elsewhere due to funding and staffing constraints, and programs elsewhere may differ from ours based on regional patient population and needs, insurance coverage, and other factors. The availability of community providers competent in military culture and trained in evidence-based treatments in this population may also affect feasibility.

## 7 Lessons learned

The clinical care model developed at Home Base has been refined over time through an iterative, flexible approach integrating patient feedback and considering clinical feasibility. While there have been many successes in our approach, we recognize several important drawbacks and ways in which our model may not be easily generalizable to other clinics. Details of these lessons learned are shared below.

### 7.1 Response to COVID-19

The challenges imposed by the COVID-19 pandemic required significant changes to the Home Base model of care. In March of 2020, Home Base rapidly pivoted to deliver its outpatient care via telehealth, which continues to be the predominant mode of treatment to date. After Massachusetts lifted the state of emergency, our patients continued to express a preference for telehealth services. The positive results of this shift have been increased access to care among veterans residing in rural areas within Massachusetts and reduced cancellation and no-show rates, including within our SOAR programming. One negative consequence of the increased use of telehealth has been technological challenges for older veterans seeking care, which is consistent with the extant literature (58). Finding the balance for our hybrid care model has been a complex task requiring calibrating clinician schedules, clinic space availability, and patient preferences. Although our services have a wider reach, we are limited to providing telehealth services only to those patients located in Massachusetts during the appointment because of licensing restrictions. Similar to models in other states such as the PsyPact, we believe new legislature is needed to increase access to telehealth care beyond state lines to bring competent, evidence-based care to veterans living in areas without access to evidence-based in-person care. Of note, when services are primarily virtual, some of the benefits of the Veteran Outreach Team as it was first conceived (e.g., in-person greetings) are missed. We continue to adapt to the changing landscape of mental health care and strive to keep our innovative programming flexible to meet the needs of our patient population.

### 7.2 An integrated model of care including family

Careful considerations must be made regarding confidentiality and privacy when treating multiple members within a family system. The current practice in our clinic is to ensure separate care teams for family members to avoid ethical conflicts. With staff providing clinical care across multiple services of the clinic, collaborative and proactive conversations are needed to ensure these ethical principles are met should multiple members of the same family present for care. Strong familiarity with HIPAA regulations and the jurisdictional ethics code should be maintained to ensure clinical information or engagement in care is not shared with other family members without consent. This knowledge should be supported by team- and institutional-based consultation when needed. Prioritizing ethical considerations and confidentiality concerns is particularly salient when families present with concerns of intimate partner violence or the safety of children or elderly members of the family. With a focused scope on supporting family members’ concerns related to their family member’s mental health, coordination with local resources is essential to ensure families can connect with resources when their concerns are outside the clinic’s scope of care.

## 8 Limitations

Home Base is a vibrant and quickly evolving program with many innovative staff members. While in many ways the passion of the team is an incredible asset (e.g., inventive new programming), it has posed challenges as well. One such challenge is the lack of consistency of our outcome measures over time as staff members have adjusted the measures based on varied programmatic and research needs. Additionally, we have missing data on follow-up measures despite our efforts to collect data regularly at predetermined intervals. For the SOAR program, data were limited by small sample sizes for post-measure completion. Recent steps toward improving the response rate to our measures have been to unify and simplify the measures collected, to increase engagement with patients and encourage their completion of the measures (e.g., individual clinicians provide reminders to patients when their measures are not complete within the desired time frame), and to improve monitoring of how these adjustments impact patient response. Some modest improvements have been noted with these concerted efforts over the past year.

Another key limitation is the potential lack of generalizability, given the characteristics of the population that we serve. The median age of all US veterans in 2018 was 65 years old. The median age of post-9/11 veterans in the same survey was 37 years old (58). The most common age group for individuals seeking care at the Home Base Outpatient Clinic is 35-49 years old; post-9/11 veterans comprise the majority of our sample. This patient selection is likely influenced by various factors. First, the Home Base Program started as a clinic focused on post-9/11 veterans and later widened its scope to include all eras. Second, Massachusetts has seen a 36% decrease in its veteran population since 2012, and approximately 35% of the veterans residing in Massachusetts are Gulf War or post-9/11 veterans (48). Our VOC teams have sought to increase outreach to older veterans and our clinic continues to seek to meet the needs of the military population in the Commonwealth, yet our patient population is not representative of the veteran population overall. A final important limitation: the Home Base partnership between Massachusetts General Hospital and the philanthropic Red Sox Foundation is a unique and invaluable collaboration that provides both institutional and financial support to sustain the work that the clinic does. However, it is unlikely to be replicated in other settings, creating further complexity in replicating our programming.

We recognize that our current clinic’s operations have arisen in a manner that may be relatively unique and therefore not fully replicable. Three key driving factors toward our present state have been: 1) the slow and deliberate progress led by a multidisciplinary leadership team over fifteen years as our program has evolved to meet our mission more effectively; 2) natural growth from leveraging the interests and skills of our clinical team (e.g., creating family and dual-diagnosis specific programming); and 3) pressure from external factors (i.e., COVID-19 pandemic) that necessitated shifts in our operational procedures. Despite the unique history of our program that was shaped by regional contextual and temporal factors, we hope our model may influence the growth of competent, evidence-based, and culturally sensitive programs for veterans, service members, and their families where collaborative relationships (internal and external), capable personnel, and commitment toward a focused goal make that possible.

## Data availability statement

The original contributions presented in the study are included in the article/supplementary material. Further inquiries can be directed to the corresponding author.

## Ethics statement

Ethical review and approval was not required for the study of human participants in accordance with the local legislation and institutional requirements. Written informed consent from the participants was not required to participate in this study in accordance with the national legislation and the institutional requirements.

## Author contributions

GH: Writing – review & editing, Writing – original draft, Supervision, Conceptualization. AT: Writing – review & editing, Writing – original draft. RL: Writing – original draft. PW: Writing – original draft. RZ: Writing – review & editing, Writing – original draft. WT: Writing – review & editing, Writing – original draft. WS: Writing – original draft. KE: Writing – original draft. JB: Writing – review & editing, Writing – original draft. KD: Writing – review & editing, Writing – original draft. JC: Writing – original draft. KT: Writing – review & editing, Writing – original draft, Supervision, Conceptualization.
